# First record of monogenean fish parasites in the Upper Lufira River Basin (Democratic Republic of Congo): dactylogyrids and gyrodactylids infesting *Oreochromis mweruensis*, *Coptodon rendalli* and *Serranochromis macrocephalus* (Teleostei: Cichlidae)

**DOI:** 10.1186/s13071-022-05637-5

**Published:** 2023-02-02

**Authors:** Gyrhaiss Kapepula Kasembele, Auguste Chocha Manda, Emmanuel Abwe, Antoine Pariselle, Fidel Muterezi Bukinga, Tine Huyse, Michiel Willem Paul Jorissen, Emmanuel Jean Willem Michel Nzambemalamu Vreven, Wilmien Jacoba Luus-Powell, Willem Johannes Smit, Joseph Roderick Sara, Jos Snoeks, Maarten Pieterjan Maria Vanhove

**Affiliations:** 1 Unité de Recherche en Biodiversité et Exploitation durable des Zones Humides (BEZHU), Faculté des Sciences Agronomiques, Université de Lubumbashi, Haut-Katanga, Democratic Republic of Congo; 2 Department of Biology, Royal Museum for Central Africa, Leuvensesteenweg 13, 3080 Tervuren, Belgium; 3 CNRS, IRD, Institut des Sciences de l’Évolution Montpellier (ISEM), Université Montpellier, Montpellier, France; 4 ISEM, Univ Montpellier, CNRS, IRD, Montpellier, France; Faculty of Sciences, Mohammed V University in Rabat, Rabat, Morocco; 5 Section de Parasitologie, Département de Biologie, Centre de Recherche en Hydrobiologie, Uvira, Democratic Republic of Congo; 6 Laboratory of Biodiversity and Evolutionary Genomics, Department of Biology, University of Leuven, Ch. Deberiotstraat 32, 3000 Leuven, Belgium; 7 Research Group Zoology: Biodiversity & Toxicology, Centre for Environmental Sciences, Hasselt University, 3590 Diepenbeek, Belgium; 8 DSI-NRF SARChI Chair, Department of Biodiversity, University of Limpopo, Sovenga, 0727 South Africa; 9 Capacities for Biodiversity and Sustainable Development, Operational Directorate Natural Environment, Royal Belgian Institute of Natural Sciences, Vautierstraat 29, 1000 Brussels, Belgium; 10 Zoology Unit, Finnish Museum of Natural History, University of Helsinki, P.O. Box 17, 00014 Helsinki, Finland

**Keywords:** Lake Tshangalele, Haut-Katanga, *Cichlidogyrus*, *Enterogyrus*, *Gyrodactylus*, *Scutogyrus*

## Abstract

**Background:**

Monogenean parasites have never been formally reported on fishes from the Lufira River Basin. In this context, we decided to record the monogenean parasite fauna of three cichlid species found in the Upper Lufira River Basin for the first time by inventorizing their diversity (species composition) and analysing their infection parameters (prevalence, mean intensity and abundance).

**Methods:**

The African cichlid fishes *Oreochromis mweruensis*, *Coptodon rendalli* and *Serranochromis macrocephalus* were selected for the study, given their economic value and their abundance in the Upper Lufira River Basin. Monogeneans were isolated from the gills and stomach, mounted on glass slides with either Hoyer’s medium or ammonium picrate-glycerin for identification under a stereomicroscope, based on morphological analysis of genital and haptoral hard parts. Indices of diversity and infections parameters were calculated.

**Results:**

A total of 13 gill monogenean parasite species (*Cichlidogyrus dossoui*, *C. halli*, *C. karibae*, *C. mbirizei*, *C. papernastrema*, *C. quaestio*, *C. sclerosus*, *C. tiberianus*, *C. tilapiae*, *C. zambezensis*, *Scutogyrus gravivaginus*, *S.* cf. *bailloni* and *Gyrodactylus nyanzae*) and one stomach monogenean (*Enterogyrus malmbergi*) were identified. A species richness (*S*) of 10 for *O. mweruensis*, *S* = 6 for *C. rendalli* and *S* = 2 for *S. macrocephalus* was recorded. Five parasite species were reported to be common amongst *O. mweruensis* and *C. rendalli.* According to cichlid species, the most prevalent parasite species was *C. halli* (prevalence [*P*] = 80.9%) on *O. mweruensis*, *C. dossoui* (*P* = 92.9%) on *C. rendalli* and *C. karibae* and *C. zambezensis* (both *P* = 9.1%) on *S. macrocephalus.* The parasite species with the highest mean intensity (MI) were *G. nyanzae* (MI = 8.7) on *O. mweruensis*, *C. papernastrema* (MI = 17.1) on *C. rendalli* and *C. karibae* (MI = 15) on *S. macrocephalus.* The findings indicate new host ranges for five parasites species (*C. quaestio, S.* cf. *bailloni, E. malmbergi* on *O. mweruensis, C. halli* on *C. rendalli* and *C. karibae* on *S. macrocephalus*) as well as new geographical records for all of them as they are recorded for the first time in the Lufira River Basin.

**Conclusions:**

This study highlighted the richness of monogenean communities in the Upper Lufira River Basin and is a starting point for future helminthological studies, such as on the use of fish parasites as indicators of anthropogenic impacts.

**Graphical Abstract:**

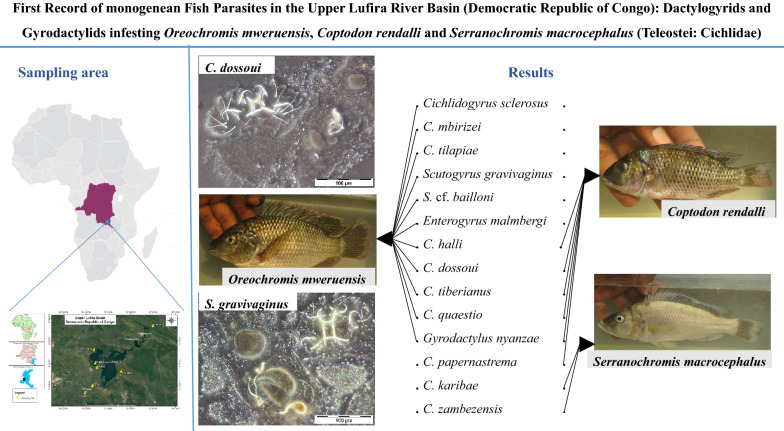

**Supplementary Information:**

The online version contains supplementary material available at 10.1186/s13071-022-05637-5.

## Background

The Congo Basin harbours the greatest species richness of fishes across the African continent [1]. The Congo Basin encompasses 3,747,320 km^2^, with a drainage area that covers most of the Democratic Republic of Congo and parts of some of its bordering countries (Angola, Zambia, Tanzania, Burundi, Rwanda, Central African Republic and Republic of Congo) and a small part of Cameroon [2]. Many different types of habitats are found in the Congo Basin, and these are subdivided into separate drainages: the Upper Congo (called Lualaba), the Middle Congo and the Lower Congo [1, 3, 4]. One of the major tributaries in the Upper Congo drainage is the Lufira River [5], which can also be subdivided into three sections: the Upper Lufira (from the source of the river to Lake Koni), the Middle Lufira (from downstream Lake Koni to the Kyubo Falls) and the Lower Lufira (from downstream the Kyubo Falls to the Kamalondo Depression, at the junction with the Lualaba River) [4, 6]. During the first half of the 20th century two successive dams were built in the Upper Lufira River to provide hydroelectric power, resulting in the creation of two artificial Lakes, Tshangalele (1930) and Koni (1949) [7–9]. Lake Tshangalele, located about 35 km east of the town of Likasi, is home to a variety of fish species, and it is also an UNESCO Biosphere Reserve, rich in bird life [10, 11]*.* Most of the studies on biodiversity undertaken to date in the Lufira River have focussed on vertebrates, such as fishes and birds [12–15]. Vast and speciose communities, which are often dominated by less sizeable animals such as flatworms or various parasite taxa, remain understudied [16, 17]. In view of the high diversity of potential host species in the tropics, it can be expected that parasitological surveys there would lead to the discovery of many parasite species, including species new to science [18, 19].

The focus of this study was monogenean fish parasites, which due to their diversity, wide distribution, high host specificity and single-host life-cycle are interesting models for studying the extent of parasite biodiversity and the underlying diversification mechanisms [20]. Monogeneans are common parasitic flatworms (Platyhelminthes) that mostly infest fish but sporadically infect aquatic invertebrates, amphibians, reptiles and a single mammalian species (the hippopotamus) [21–26]. Parasitic monogeneans present a high risk for aquaculture industries, causing substantial economic losses, and have been associated with reduced growth, morbidity and mortality [27–29]. Several monogenean species are reported to have serious economic impacts in the confines of captive or intensive fish farming [30, 31].

The infection sites of monogeneans on fish hosts are typically gills, fins and/or skin [32]; however, very occasionally they are also found in the stomach, urinary bladder, intestine, oral or nasal cavity, eyes and heart [33, 34]. Due to a one-host life-cycle and a close relationship with the respective host species, many monogeneans are specialists, infesting only a single host species (oioxenous specificity), although others are generalists, infesting ≥ 2 host species (stenoxenous specificity) [35–37]. Mendlová and Šimková [38] used a more extensive number of categories of host specificity on the basis of the phylogenetic relationships among (cichlid) host species in which parasites can be: (i) strict specialists when infecting only one host species; (ii) intermediate specialists when infecting ≥ 2 congeneric host species; (iii) intermediate generalists when infecting noncongeneric cichlid species belonging to the same tribe; and finally (iv) generalists, when infecting noncongeneric cichlid species of at least two different tribes.

African cichlids (taking also into account the Levant) are known to harbour monogenean parasites belonging to six genera: *Enterogyrus* Paperna, 1963; *Urogyrus* Bilong Bilong, Birgi & Euzet, 1994; *Onchobdella* Paperna, 1968; *Scutogyrus* Pariselle & Euzet, 1995; *Cichlidogyrus* Paperna, 1960 (Dactylogyridea) and *Gyrodactylus* von Nordmann, 1832 (Gyrodactylidea). The latter four are ectoparasitic genera, and among these, *Cichlidogyrus* is the most species-rich group with more than 131 nominal species described to date [39–41]. Its representatives are known to be pathogenic in tilapia aquaculture [42].

The overall aim of this study was to record the monogenean parasite fauna of three cichlid species found in the Upper Lufira River Basin. At the start of this study, these parasites had never been formally reported from this region. The specific objectives include: (i) inventorizing the diversity of gill monogenean communities, and (ii) analysing infection parameters of these monogenean parasites.

## Methods

### Study area

**Fig. 1:**
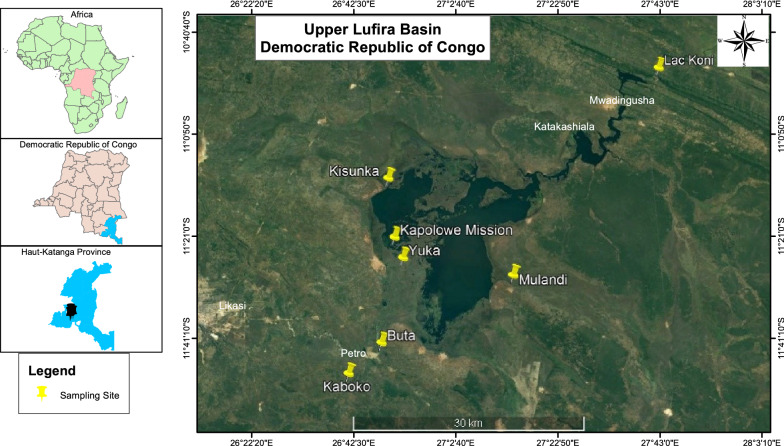
Map of sampling sites in the Upper Lufira River Basin: along the Lufira River (Kaboko: 11°4′31.60″ S, 26°55′2.40″ E; Buta: 11°2′21.60″ S, 26°57′23.10″ E) and bordering two stretches of the Lufira River that have been dammed: Lake Tshangalele (Kisunka: 10°50′52.10″ S, 26°57′50.60″ E; Kapolowe Mission: 10°54′59.50″ S, 26°58′17.70″ E; Yuka: 10°56′25.30″ S, 26°58′53.40″ E; Mulandi: 10°57′36.64″ S, 27°6′44.88″ E) and Lake Koni (Koni: 10°43′3.65″ S, 27°17′3.24″ E)

This study was conducted in the Upper Lufira River Basin (altitude: 1114–1160 m a.s.l.) (Fig. 1), which is localized across the mining hinterland area in the west of Haut-Katanga Province (in the south of the former Katanga Province). The climate type is equatorial savanna with a dry winter (type AW6: A, equatorial climate region; W, desert) following the classification of Köppen [43] and a rainy tropical climate with a rainy season extending from November to April [44]. Most precipitation falls from December to March [45]. The Upper Lufira River Basin is characterized by a great diversity of fish species, including members of the families Alestidae, Anabantidae, Amphilidae, Auchenoglanididae, Characidae, Clariidae, Cichlidae, Cyprinidae, Cyprinodontidae, Distichodontidae, Mochokidae, Mormyridae and Schilbeidae [46, 47]. There is organized small-scale fishing for *Coptodon rendalli* (Boulenger, 1896), *Oreochromis mweruensis* Trewavas, 1983, *Serranochromis macrocephalus* Boulenger, 1899, *Clarias gariepinus* (Burchell, 1822) and *Clarias ngamensis* (Castelnau, 1861) [48]. Caught fishes are intended for human consumption, for a small part by the local population around the Upper Lufira River Basin and for most part by residents of bigger towns, such as Likasi and Lubumbashi.

### Fish sampling

Three fish species, *O. mweruensis*, *C. rendalli* and *S. macrocephalus* were selected for examination in the study, given their economic value and their abundance in the Upper Lufira River Basin [48]. Fishes were collected using gillnets with a mesh size of 12–20 mm knot to knot or were bought from local fishermen along the shores of the Lufira River, Lake Tshangalele and Lake Koni (Fig. 1) between September 2015 and August 2018. Once obtained, the fishes were kept alive in an aerated tank and transported to a field laboratory where they were identified to the species level following the keys of Lamboj [47]. The fishes were killed by severing the spinal cord just posterior to the cranium, immediately prior to examination, following the method of Olivier et al. [49]. For each fish, total length (TL) and standard length (SL) were measured to the nearest 0.1 cm, and the weight was taken to the nearest 0.1 g.

### Parasite sampling

To collect monogenean parasites, we first dissected the fishes and removed the right gill arches by dorso-ventral section. One fish specimen from amongst all of the fish species sampled was randomly dissected and inspected for monogenean parasites in its stomach. Gill arches and the stomach were placed in a Petri dish containing water for examination using an (Optika 4.0.0 stereomicroscope (OPTIKA Srl, Ponteranica, BG, Italy). Parasites were dislodged from the gill filaments using entomological needles and fixed between a slide and coverslip into a drop of either Hoyer’s medium or ammonium picrate-glycerin, according to Nack et al*.* [50]. After 24 h, the coverslips were sealed with nail varnish. Parasites were deposited in the invertebrate collection of the Royal Museum of Central Africa (RMCA) under accession numbers RMCA_VERMES_43743-44345.

### Monogenean community composition, indices of diversity and infection parameters

Morphological identification of the retrieved parasite specimens was conducted based on the sclerotized parts of the haptor, the male copulatory organ and the vagina, using a Motic BA310 microscope (Motic, Speed Fair Co., Ltd., Hong Kong) and a phase-contrast microscope (model BX50; Olympus, Tokyo, Japan). Parasite identification to species level and comparison with known congeners was based on García-Vásquez et al*.* [51, 52], Přikrylová et al*.* [53, 54], Gillardin et al*.* [55], Muterezi Bukinga et al*.* [56], Pariselle and Euzet [39, 57] and Fannes et al*.* [58]. Parasite diversity was summarized by the species richness index, Shannon index and Equitability of Pielou [59]. Infection parameters, i.e. prevalence, mean intensity (MI) and mean abundance (MA) were provided following definitions given by Margolis et al*.* [60] and Bush et al*.* [61]. Statistical analysis was performed using Past version 3.1 software [62].

## Results

Fishes used in this study were of different sizes and weight ranges. For *O. mweruensis* (*n* = 47), the mean (± standard deviation) TL and SL were 18.2 ± 4.1 and 14.6 ± 3.2 cm, respectively, and the mean weight was 72.7 ± 38.8 g. For *C. rendalli* (*n* = 28), the mean TL and SL were 15.1 ± 2.8 and 12.0 ± 2.4 cm, respectively, and the mean weight was 72.7 ± 38.8 g. For *S. macrocephalus* (*n* = 11), the mean TL and SL were 16.9 ± 3.4 and 14.0 ± 2.8 cm, respectively, and the mean weight was 81.9 ± 51.5 g.

### Composition and indices of diversity of the Monogenean community in the Upper Lufira River Basin

**Fig. 2:**
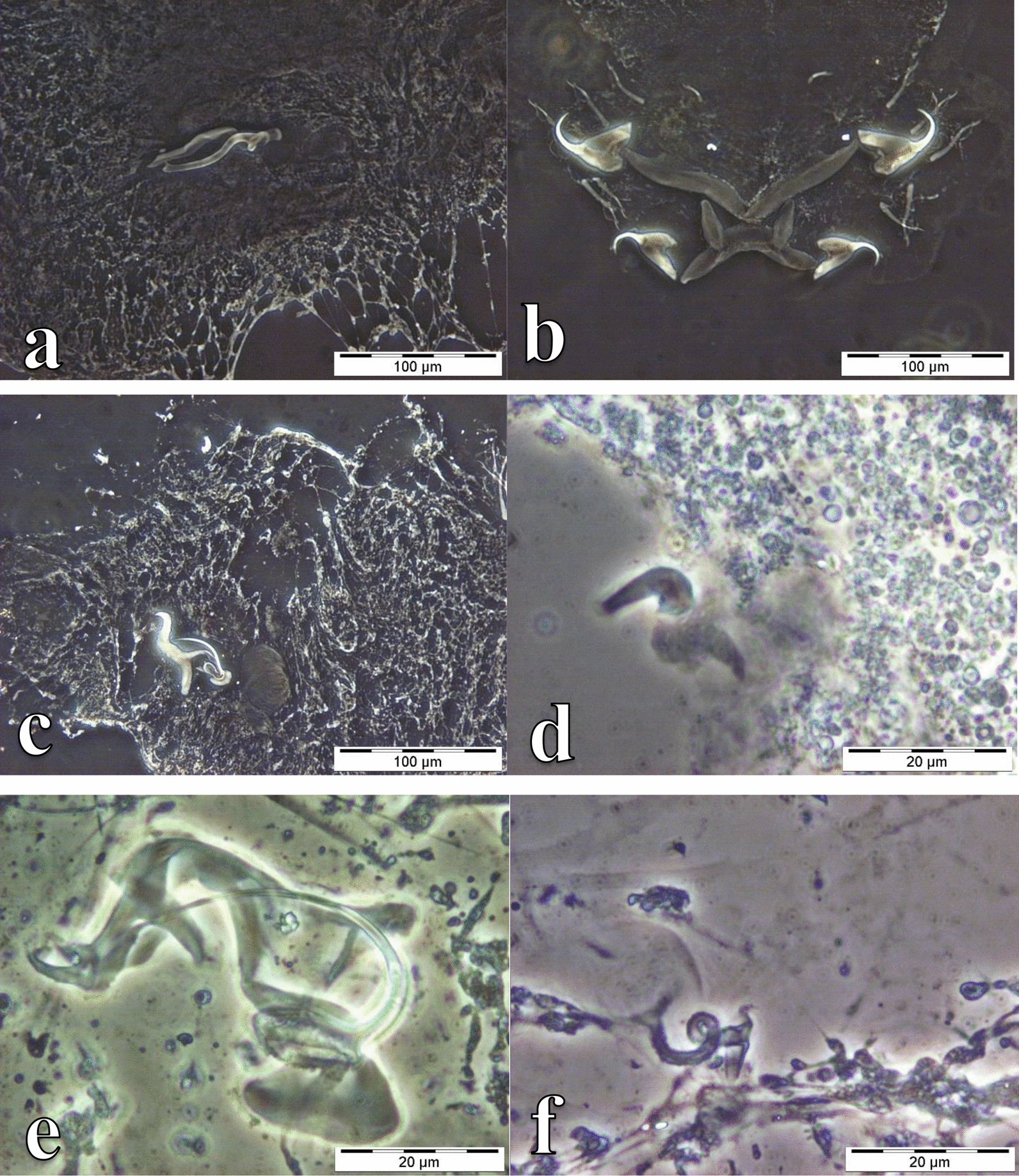
Photomicrographs of the sclerotized structures of:** a** the male copulatory organ of *Cichlidogyrus halli* ex *Oreochromis mweruensis* from Lake Koni (RMCA_VERMES_44100), **b** the haptor of *C. halli* ex *O. mweruensis* from Lake Koni (RMCA_VERMES_44101), **c** the male copulatory organ of *Cichlidogyrus dossoui* ex *Coptodon rendalli* from Lufira River (RMCA_VERMES_43783), **d** the vagina of *C. dossoui* ex *C. rendalli* from Yuka (RMCA_VERMES_44286), **e** the male copulatory organ of *Cichlidogyrus tiberianus* ex *C. rendalli* from Kapolowe Mission (RMCA_VERMES_44026), **f** the vagina of *C. tiberianus* ex *C. rendalli* from Kapolowe Mission (RMCA_VERMES_43997)

**Fig. 3:**
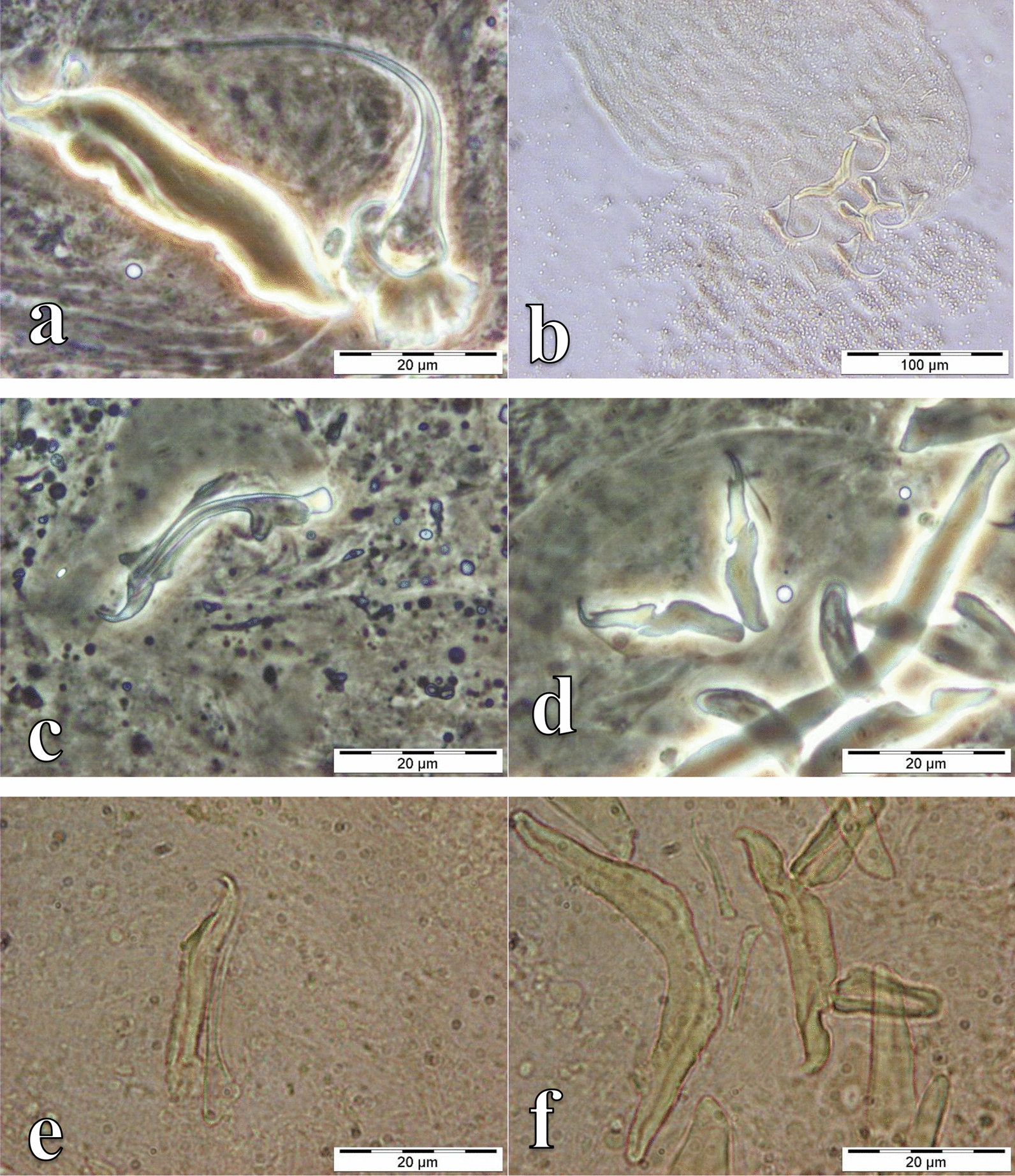
photomicrographs of the sclerotized structures of:** a** the male copulatory organ of *Cichlidogyrus sclerosus* ex *Oreochromis mweruensis* from Buta (RMCA_VERMES_43900), **b** the haptor of *C. sclerosus* ex *O. mweruensis* from Kisunka (RMCA_VERMES_43902), **c** the male copulatory organ of *Cichlidogyrus quaestio* ex *Coptodon rendalli* from Kisunka (RMCA_VERMES_43934), **d** the haptor of *C. quaestio* ex *C. rendalli* from Kisunka (RMCA_VERMES_43934), **e** the male copulatory organ of *Cichlidogyrus tilapiae* ex *O. mweruensis* from Kisunka (RMCA_VERMES_43910), **f** the haptor of *C. tilapiae* ex *O. mweruensis* from Kisunka (RMCA_VERMES_43910)

**Fig. 4:**
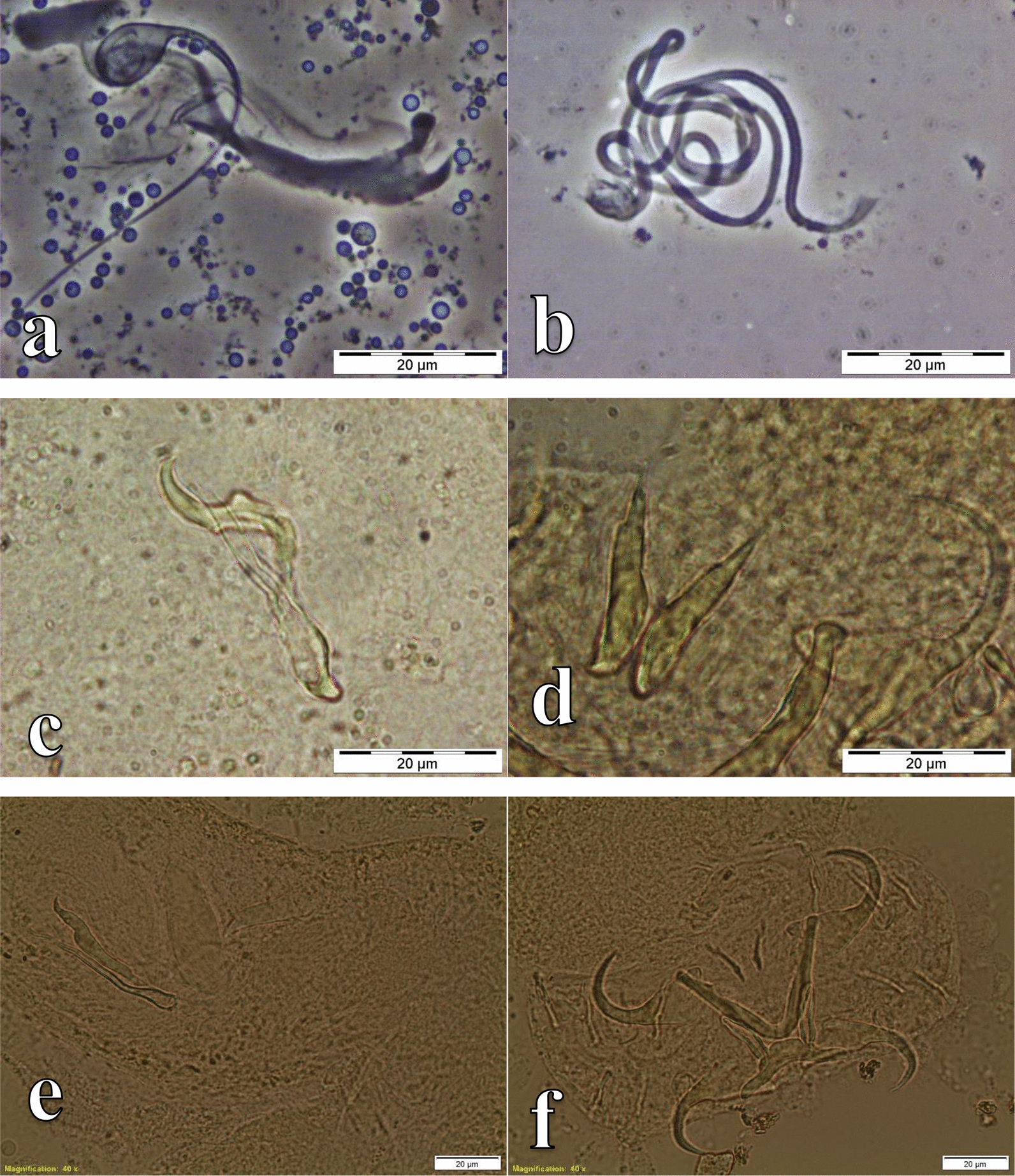
Photomicrographs of the sclerotized structures of:** a** the male copulatory organ of *Cichlidogyrus mbirizei* ex *Oreochromis mweruensis* from Lufira River (RMCA_VERMES_43753), **b** the vagina of *C. mbirizei* ex *O. mweruensis* from Lufira River (RMCA_VERMES_43766), **c** the male copulatory organ of *Cichlidogyrus papernastrema* ex *Coptodon rendalli* from Lufira River (RMCA_VERMES_43817), **d** the first pair of marginal hooks of *C. papernastrema* ex *C. rendalli* from Lufira River (RMCA_VERMES_43791), **e** the male copulatory organ of *Cichlidogyrus karibae* ex *Serranochromis macrocephalus* from Lufira River (RMCA_VERMES_44341)*,* **f** the haptor of *C. karibae* ex *S. macrocephalus* from Lufira River (RMCA_VERMES_44341)

**Fig. 5:**
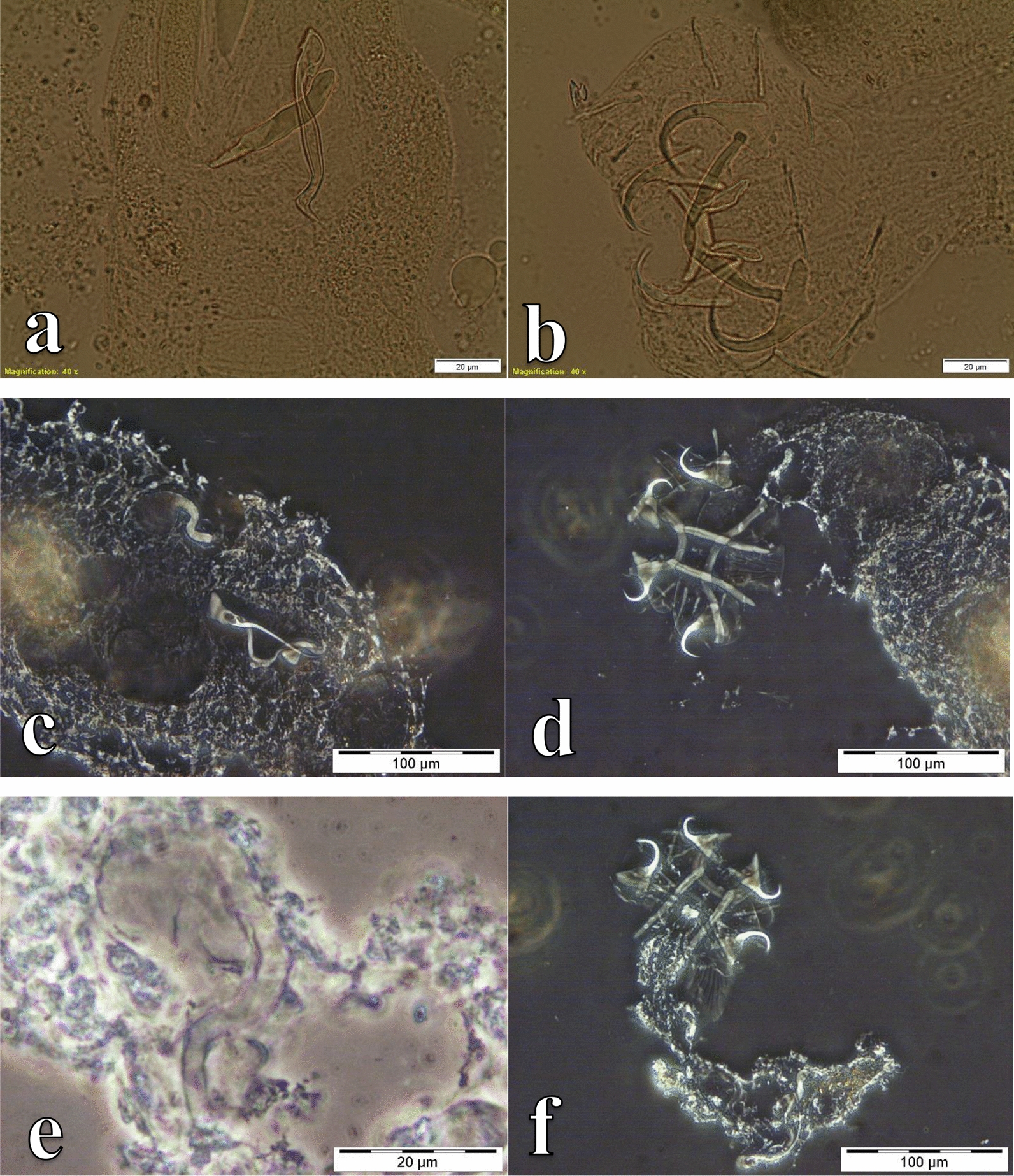
Photomicrographs of the sclerotized structures of:** a** the male copulatory organ of *Cichlidogyrus zambezensis* ex *Serranochromis macrocephalus* from Lufira River (RMCA_VERMES_44343), **b** the haptor of *C. zambezensis* ex *S. macrocephalus* from Lufira River (RMCA_VERMES_44343), **c** the male copulatory organ and vagina of *Scutogyrus gravivaginus* ex *Oreochromis mweruensis* from Buta (RMCA_VERMES_43896), **d** the haptor of *S. gravivaginus* ex *O. mweruensis* from Buta (RMCA_VERMES_43896), **e** the vagina of *Scutogyrus* cf. *bailloni* ex *O. mweruensis* from Kapolowe Mission (RMCA_VERMES_43958), **f** the whole mount of *Scutogyrus* cf. *bailloni* ex *O. mweruensis* from Kapolowe Mission (RMCA_VERMES_43958)

**Fig. 6:**
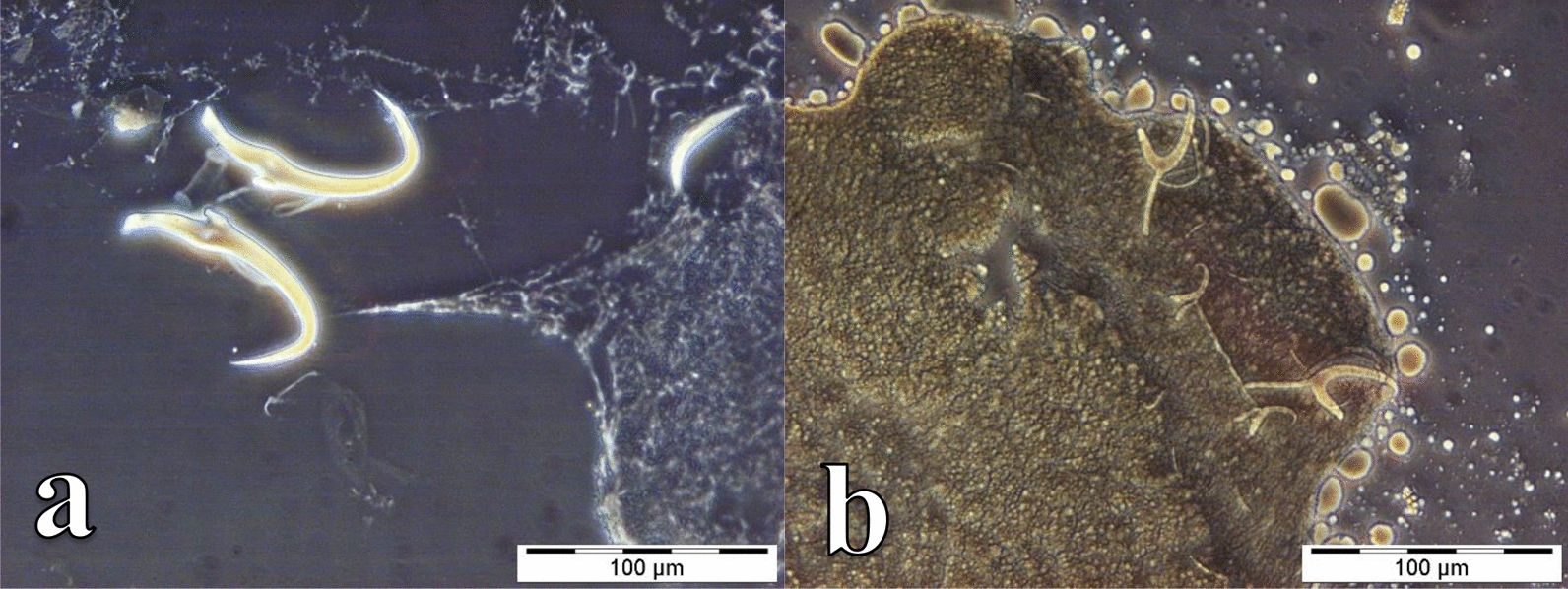
Photomicrographs of the sclerotized structures of:** a** the haptor of *Gyrodactylus nyanzae* ex *Oreochromis mweruensis* from Lufira River (RMCA_VERMES_43758), **b** the haptor of *Enterogyrus malmbergi* ex *O. mweruensis* from Lake Koni (RMCA_VERMES_44106)

Specimens representing four genera of monogeneans, *Cichlidogyrus, Gyrodactylus*, *Scutogyrus* (on the gills) and *Enterogyrus* (in the stomach) were collected (Table 1; Additional file 1: Table S1). Among these were 10 known species of *Cichlidogyrus*, one species of *Gyrodactylus*, two species of *Scutogyrus* and one species of *Enterogyrus* (Figs. 2, 3, 4, 5, and 6). For *O. mweruensis*, *C. rendalli* and *S. macrocephalus*, the parasite diversity indices were, respectively, 10, 6 and 2 for the species richness index; 1.5, 1.2 and 0.6 for the Shannon index; and 0.6, 0.8 and 0.8 for the index of Equitability of Pielou. The distribution of monogeneans per sampling period or per season is shown in Table 2 to provide a picture of the distribution of monogeneans over time.

**Table 1:** The monogenean parasite species recovered from *Oreochromis mweruensis*, *Coptodon rendalli* and *Serranochromis macrocephalus* in the Upper Lufira River Basin

Parasite order	Parasite genus	Parasite species	Host species	No. of hosts examined	No. of hosts infected
Dactylogyridea Bychowsky, 1937	*Cichlidogyrus* Paperna, 1960	*C. halli* (Price & Kirk, 1967)	*Oreochromis mweruensis*	45	39
			*Coptodon rendalli*	29	4
		*C. dossoui* Douëllou, 1993	*O. mweruensis*	45	19
			*C. rendalli*	29	25
		*C. sclerosus* Paperna & Thurston, 1969	*O. mweruensis*	45	14
		*C. tiberianus* Paperna, 1960	*O. mweruensis*	45	1
			*C. rendalli*	29	12
		*C. quaestio* Douëllou, 1993	*O. mweruensis*	45	2
			*C. rendalli*	29	13
		*C. mbirizei* Muterezi Bukinga, Vanhove, Van Steenberge & Pariselle, 2012	*O. mweruensis*	45	9
		*C. tilapiae* Paperna, 1960	*O. mweruensis*	45	7
		*C. papernastrema* Price, Peebles & Bamford, 1969	*C. rendalli*	29	15
		*C. karibae* Douëllou, 1993	*Serranochromis macrocephalus*	11	1
		*C. zambezensis* Douëllou, 1993	*S. macrocephalus*	11	1
	*Enterogyrus* Paperna, 1963	*E. malmbergi* Bilong Bilong, 1988	*O. mweruensis*	1	1
	*Scutogyrus* Pariselle and Euzet, 1995	*S. gravivaginus* (Paperna & Thurston, 1969)	*O. mweruensis*	45	20
		*S.* cf. *bailloni* Pariselle & Euzet, 1995	*O. mweruensis*	45	1
Gyrodactylidea Bychowsky, 1937	*Gyrodactylus* Von Nordmann, 1832	*G. nyanzae* Paperna, 1973	*O. mweruensis*	45	12
			*C. rendalli*	29	1

**Table 2:** The average number of parasites per infected fish species per host individual according to sampling period

Monogenean species	Sampling date^a^				
	September. 2015	March 2016	April 2016	August 2016	September 2017
*Oreochromis mweruensis*					
*Cichlidogyrus dossoui*	1.4	5.7	3.1	13	5.3
*C. halli*	3.6	10.2	6.1	1.5	13.6
*C. mbirizei*			1		2
*C. quaestio*	1		1		
*C. sclerosus*	2	1	1.3		2.6
*C. tiberianus*			1		
*C. tilapiae*	1	2	1		1
*Gyrodactylus nyanzae*	6.5		23		9.6
*Scutogyrus gravivaginus*	1.3	1.7	4.7	3.5	1.8
*S.* cf. *bailloni*			1		
Average total number of monogeneans, all species included	16.8	20.6	43.2	18	35.9
Number of examined fish	12	5	13	2	13
*Coptodon rendalli*					
*Cichlidogyrus dossoui*	5.1	7.3	21.3	15	2
*C. halli*	1		2		
*C. papernastrema*	1.2		29.8	25	2.5
*C. quaestio*	5.4	7	11		1
*C. tiberianus*	1.3	1.5	4.4	1.5	
*Gyrodactylus nyanzae*	1				
Average total number of monogeneans, all species included	15	15.8	68.5	41.5	5.5
Number of examined fish	10	6	8	2	3

Monogenean species	Sampling date				
	September 2017	August 2018			
*Serranochromis macrocephalus*					
*Cichlidogyrus karibae*	15				
*C. zambezensis*	5				
Average total number of monogeneans, all species included	20				
Number of examined fish	1	10			

^a^August, September are the dry season; March and April are the rainy season

### Infection parameters of monogenean parasites in the Upper Lufira River Basin

The prevalence, mean intensity and mean abundance presented here take into account conspecific host individuals grouped without seasonal subdivision as the main objective of the study was to record the monogenean parasite diversity, not the epidemiological variation.

**Fig. 7:**
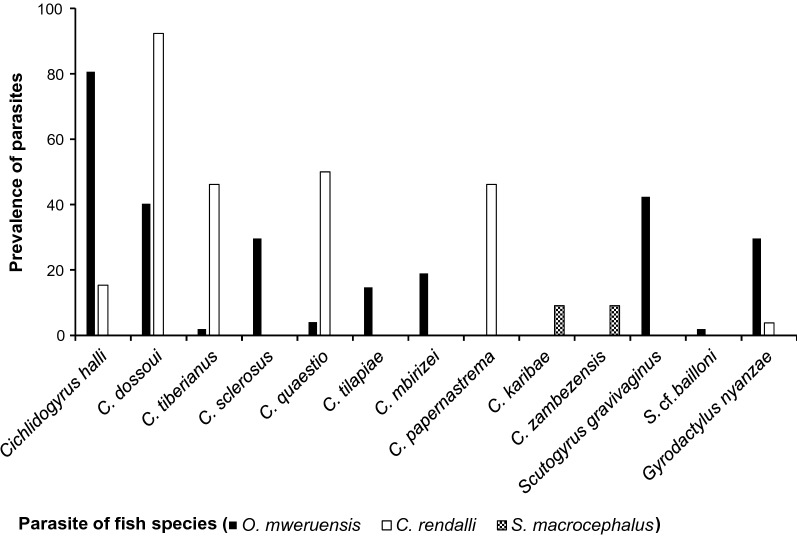
Parasite prevalence (%) per monogenean species recovered on the gills of *Oreochromis mweruensis*, *Coptodon rendalli* and *Serranochromis macrocephalus* in the Upper Lufira River Basin

The highest prevalences recorded were 80.9% for *Cichlidogyrus halli* on *O. mweruensis*, 92.3% for *Cichlidogyrus dossoui* on *C. rendalli* and 9.1% for both *Cichlidogyrus zambezensis* and *Cichlidogyrus karibae* on *S. macrocephalus.* The lowest prevalences recorded were 2.1% for *Cichlidogyrus tiberianus*, *Scutogyrus* cf. *bailloni* on *O. mweruensis* and 3.8% for *Gyrodactylus nyanzae* on *C. rendalli* (Fig. 7)*.*

**Fig. 8:**
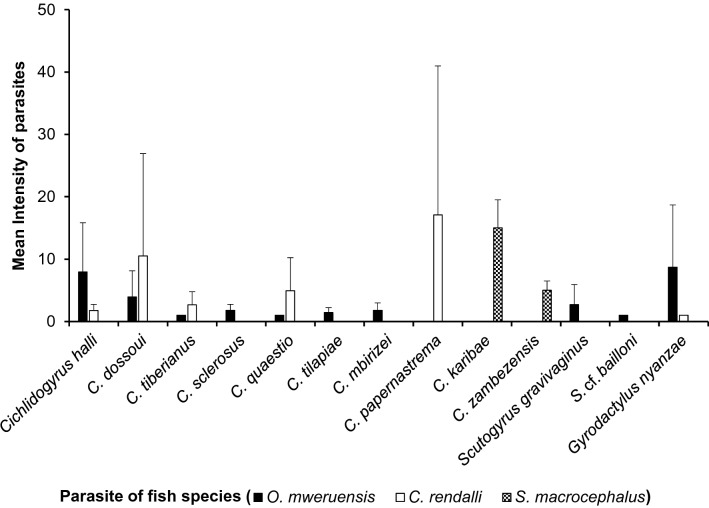
Mean intensity of each monogenean species recovered on the gills of *Oreochromis mweruensis*, *Coptodon rendalli* and *Serranochromis macrocephalus* in the Upper Lufira River Basin. Whisker above the mean indicates the standard deviation

For *G. nyanzae*, the highest mean intensity of 8.7 ± 9.9 was recorded from *O. mweruensis* and the lowest mean intensity of 1 ± 0 was recorded from *C. rendalli.* Conversely, the mean intensity for *Cichlidogyrus papernastrema* was 17.1 ± 24 when the latter fish host was examined. From *S. macrocephalus*, the highest and lowest mean intensities were for *C. karibae* (MI = 15) and *C. zambezensis* (MI = 5), respectively (Fig. 8).

**Fig. 9:**
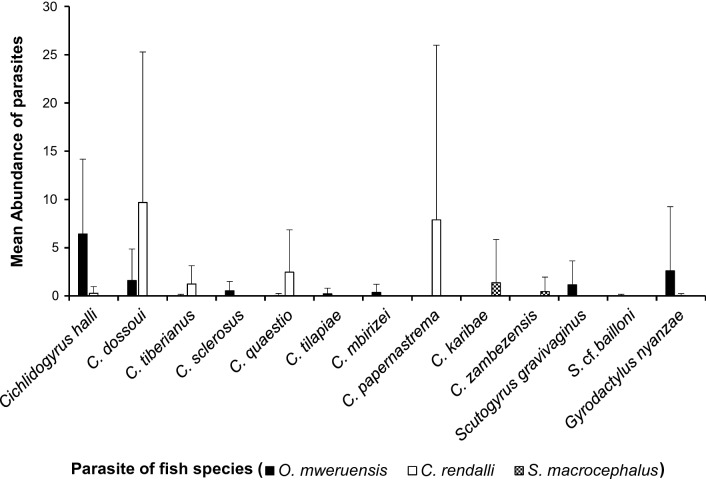
Mean abundance of each monogenean species recovered on the gills of *Oreochromis mweruensis*, *Coptodon rendalli* and *Serranochromis macrocephalus* in the Upper Lufira River Basin, with standard deviation

Results on the mean abundance revealed that *C. halli* was the most abundant species on *O. mweruensis* (MA = 6.4 ± 7.7), *C. dossoui* was the most abundant species on the gills of *C. rendalli* (9.7 ± 15.6) and *C. karibae* was the most abundant species on *S. macrocephalus* (MA = 1.4 ± 4.5) (Fig. 9).

## Discussion

This study was conducted to explore the monogenean parasite fauna of three economically important and abundant cichlid species in the Upper Lufira River Basin, a part of the Upper Congo Basin. During this study we recorded 13 gill and one stomach monogenean species. Parasite species from fish species belonging to the genera *Oreochromis* Günther, 1889*, Coptodon* Gervais, 1853 and *Serranochromis* Regan, 1920 have been previously reported [39, 56, 63]. Although a few studies on monogenean parasites from the Congo Basin have been conducted in the Lake Tanganyika, Bangweulu-Mweru, Upper Lualaba, Kasai, Lower Congo and Pool Malebo Ecoregions (sensu Thieme et al. [64]) [55, 56, 63, 65–68], the present study is the first to record monogenean parasites in the Lufira River Basin. Based on the results of previous studies and current information, this study extends the known host range of five parasite species. *Cichlidogyrus quaestio*, *S.* cf. *bailloni* and *E. malmbergi* were recorded for the first time from *O. mweruensis*; *C. halli* was recorded for the first time from *C. rendalli*; and *C. karibae* was recorded for the first time from *S. macrocephalus*. *Cichlidogyrus karibae* was described by Douëllou [69] on *Sargochromis codringtonii* (Boulenger, 1908) in Lake Kariba (Zambezi Basin, Zimbabwe). *Enterogyrus malmbergi* was described by Bilong Bilong [70] from the stomach of *Oreochromis niloticus* (Linnaeus, 1758) in the Sanaga River (Cameroon). *Scutogyrus bailloni* was formally described by Pariselle and Euzet [57] on *Sarotherodon galilaeus* (L, 1758) in the Mékrou River (Niger Basin, Niger, West Africa). Since only a single similar parasite specimen was retrieved in the present study on the gills of *O. mweruensis*, it cannot be assigned to *S. bailloni* with certainty as the mount was imperfect, although we were able to recognize and identify the principal diagnostic structures (the haptor, the male copulatory organ and the vagina). Verification of its identification with molecular markers is necessary to determine whether this specimen belongs to *S. bailloni* or to a morphologically similar species currently unknown to science. Nevertheless these (putative in case of *S. bailloni*) records substantially expand the known geographical distribution of these three monogenean species, as this study is the first time they have been recorded in the Congo Basin.

In terms of species richness, our results are similar to those reported earlier for monogenean gill parasites on these three fish species in the Congo Basin [63, 66, 68]. In the present study, 10 monogenean species were found on *O. mweruensis*, while Jorissen et al*.* [63, 66] collected nine parasite species in the Bangweulu-Mweru Ecoregion on *O. mweruensis* (of which 7 were shared, with the exceptions of *Cichlidogyrus mbirizei*, *C. quaestio* and *S.* cf. *bailloni* on *O. mweruensis* from the Lufira River system, and *C. cirratus* and *C*. *papernastrema* on *O. mweruensis* from the Bangweulu-Mweru Ecoregion)*.* Six monogenean species were found on *C. rendalli* in the present study, while Jorissen et al*.* [63, 66] collected five parasite species (all but *C. halli* corresponding to those found in this study) in the Bangweulu-Mweru Ecoregion. On *S. macrocephalus*, we found two monogenean species (*C. karibae* and *C. zambezensis*), while Jorissen et al*.* [66] reported only the latter species on *S. macrocephalus* and its congeners *Serranochromis thumbergi* (Castenau, 1861), *Serranochromis jallae* (Boulenger, 1896) and *Sargochromis mellandi* (Boulenger, 1905).

In terms of infection parameters, on *O. mweruensis*, one parasite species had a prevalence of > 50% in the Upper Lufira River Basin (*C. halli*, *P* = 80.9%) against two monogenean species in the Bangweulu-Mweru Ecoregion reported by Jorissen et al*.* [66] (*P* = 57.1% for *C. dossoui* and *S. gravivaginus*)*.* On *C. rendalli, C. dossoui* (*P* = 92.3%) in the Upper Lufira River Basin, and *C. dossoui, C. quaestio* and *C. tiberianus* in the Bangweulu-Mweru Ecoregion have *P* > 50% following comparison with Jorissen et al*.* [66]. On *S. macrocephalus*, no parasite species had a prevalence > 50% in the Upper Lufira River Basin, while *C. zambezensis* reached a prevalence of 100% in the Bangweulu-Mweru Ecoregion. Regarding the infection intensity (Table 1), the most infected individuals of *O. mweruensis* in the Upper Lufira River Basin harboured up to 30 specimens of *C. halli,* followed by 25 specimens of *G. nyanzae*, against 37 parasite specimens of *G. nyanzae* and 21 parasite specimens of *C. cirratus* in Bangweulu-Mweru Ecoregion (reported by Jorissen et al*.* [66]). The most infected individuals of *C. rendalli* in the Upper Lufira River Basin harboured up to 84 specimens of *C. papernastrema*, followed by *C. dossoui* with 68 monogenean specimens. On the other hand, on the same fish species, in the Bangweulu-Mweru Ecoregion, the monogeneans *C. dossoui* and *C. quaestio* reached a lower maximum intensity of infection (29 and 20 specimens, respectively). Finally, on *S. macrocephalus* in the Upper Lufira, the most infected fish specimens contained up to 15 and 5 parasite specimens of *C. karibae* and *C. zambezensis*, respectively, while Jorissen et al*.* [66] reported up to 21 parasite specimens of *C. zambezensis* on *Serranochromis* spp. in the Bangweulu-Mweru Ecoregion. These differences in infection parameters may be due to sample size, season, biogeographical distribution or other environmental parameters, as communities of cichlid-infecting monogeneans have been observed to fluctuate seasonally and between habitat types, and parasite species composition may change between areas and basins [71, 72].

## Conclusion

In this study, we recorded the species richness and infection parameters of three cichlid species in the Upper Lufira River Basin that infect the stomach and gills. A total of 13 monogenean species were recovered from *O. macrochir*, *C. rendalli* and *S. macrocephalus*. These findings are the first record of monogeneans in the Lufira River Basin. For future sampling, it will also be interesting to study groups of fish parasites other than monogenean parasites, as well as other fish species or families, to record the diversity of parasites. In many parts of the Congo Basin, there is a lack of baseline data on fish parasites. With this in mind, this study may serve as an important baseline for future studies conducted on fish from the Upper Lufira River Basin as well as the rest of the Congo Basin, enabling the comparison of values to those found in the present study to establish whether there has been a change in parasite composition and parasite load over time. In future studies, molecular analyses may be useful to confirm the morphological identification of parasites and identify phylogeographical patterns.

## Supplementary Information

**Additional file 1.** Voucher specimens of monogenean parasites available in the invertebrate collection of the Royal Museum of Central Africa, Tervuren, Belgium under accession numbers RMCA_VERMES_43743-44345.

## Data Availability

Slides of monogenean parasites are available in the invertebrate collection of the Royal Museum of Central Africa, Tervuren, Belgium under accession numbers RMCA_VERMES_43743-44345 (Additional file 1: Table S1).
